# Structural Requirements of *N*-alpha-Mercaptoacetyl Dipeptide (NAMdP) Inhibitors of *Pseudomonas Aeruginosa* Virulence Factor LasB: 3D-QSAR, Molecular Docking, and Interaction Fingerprint Studies

**DOI:** 10.3390/ijms20246133

**Published:** 2019-12-05

**Authors:** José Luis Velázquez-Libera, Juliana Andrea Murillo-López, Alexander F. de la Torre, Julio Caballero

**Affiliations:** 1 Centro de Bioinformática y Simulación Molecular (CBSM), Universidad de Talca, Casilla 747, Talca 3460000, Chile; josevlibera2010@gmail.com; 2 Departamento de Ciencias Químicas, Facultad de Ciencias Exactas, Universidad Andres Bello, Autopista Concepción–Talcahuano 7100, Talcahuano 4260000, Chile; jamurillo1987@gmail.com; 3 Departamento de Química Orgánica, Facultad de Ciencias Químicas, Universidad de Concepción, Concepción 4030000, Chile

**Keywords:** *Pseudomonas aeruginosa* elastase, N-alpha-mercaptoacetyl dipeptides, 3D-QSAR, docking, interaction fingerprints

## Abstract

The zinc metallopeptidase *Pseudomonas* elastase (LasB) is a virulence factor of *Pseudomonas aeruginosa* (*P. aeruginosa*), a pathogenic bacterium that can cause nosocomial infections. The present study relates the structural analysis of 118 *N*-alpha-mercaptoacetyl dipeptides (NAMdPs) as LasB inhibitors. Field-based 3D-QSAR and molecular docking methods were employed to describe the essential interactions between NAMdPs and LasB binding sites, and the chemical features that determine their differential activities. We report a predictive 3D-QSAR model that was developed according to the internal and external validation tests. The best model, including steric, electrostatic, hydrogen bond donor, hydrogen bond acceptor, and hydrophobic fields, was found to depict a three-dimensional map with the local positive and negative effects of these chemotypes on the LasB inhibitory activities. Furthermore, molecular docking experiments yielded bioactive conformations of NAMdPs inside the LasB binding site. The series of NAMdPs adopted a similar orientation with respect to phosphoramidon within the LasB binding site (crystallographic reference), where the backbone atoms of NAMdPs are hydrogen-bonded to the LasB residues N112, A113, and R198, similarly to phosphoramidon. Our study also included a deep description of the residues involved in the protein–ligand interaction patterns for the whole set of NAMdPs, through the use of interaction fingerprints (IFPs).

## 1. Introduction

Nosocomial infections—also known as hospital acquired infections—are general or localized infectious processes in organs or anatomical regions, acquired by patients during hospitalization or through visits to other local health care centers. These undesired infections are considered to be a big global problem due to their social and economic impact [1]. The ongoing abuse of antibiotics to treat nosocomial infections has led to the problem of multidrug resistance [1], which is one of the major challenges of antimicrobial discovery [2,3,4,5]. In the past, the assembly required for antibiotic drugs development [6,7] was aimed to obtain compounds that inactivate bacterial targets through specific mechanisms (i.e., inhibition of cell growth or causing cell death) [8]. As is known, pathogenic bacteria can produce a wide range of virulence factors that specifically participate in host functions, in order to allow colonization. Research is currently directed to seek specific antimicrobial agents focused on the action mechanisms of virulence factors [9].

*Pseudomonas aeruginosa (P. aeruginosa)* is a Gram–negative opportunistic pathogenic bacterium that is responsible for many nosocomial infections [10,11]. It has multiple virulence factors, such as the toxin metalloprotease *Pseudomonas elastase* (LasB), which is responsible for lung hemorrhages and corneal tissue destruction. Additionally, LasB can inactivate the alpha 1 proteinase inhibitor that controls tissue destruction through the degradation of a series of proteins, including elastin, collagen, and fibrin [12]. The idea that decreasing the function of virulence factors leads to less resistance of *P. aeruginosa* to antibiotics is still valid and was previously afforded [5,9]. In this context, some scientific endeavors have focused on developing new molecules that contain specific functional groups capable of interacting with the zinc ion of LasB metalloprotease [13,14,15], and additional chemical groups that are optimized to form specific interactions with the residues in the binding site of this target protein.

To support these tasks, theoretical studies oriented to characterize inhibitor interactions with the LasB crystal enzyme [16] could help with the development of new specific drugs to avoid antibiotic resistance in *P. aeruginosa*. Despite the widespread use of computational methods for drug design, there are a few studies related to the LasB inhibitors [17,18,19]. Fortunately, LasB–ligand complexes have been reported by X–ray crystallography [19]. Notwithstanding of several biological evaluations of sets of LasB inhibitors [13,14,15,17,18,20,21], a quantitative structure–activity relationship (QSAR) to predict and correlate the efficiency of the molecules reported is not present in the literature; neither is a deep description of the LasB binding site.

Inspired by the low number of theoretical studies dedicated to LasB inhibitors, we carried out QSAR and docking studies of the congeneric family of 118 *N*-alpha-mercaptoacetyl dipeptides (NAMdPs) reported by Cathcart et al. [17,20], providing interesting information about their binding poses and the causes of their differential activities. We assumed that this information could be useful for the design of new potential LasB inhibitors.

## 2. Results and Discussion

### 2.1. Results of the QSAR Models

**Figure 1:**
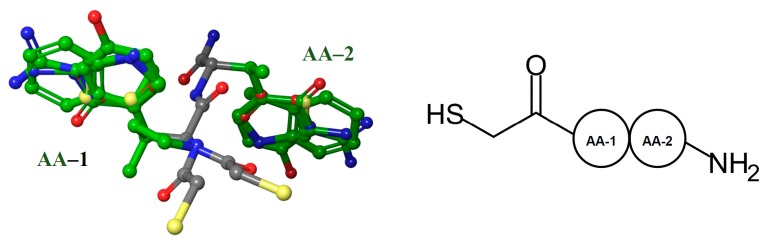
Structural alignment representation of the 118 LasB inhibitors; (left) backbone carbon atoms in gray and side chain carbon atoms in green, and (right) schematic structure of the NAMdPs.

First, the 118 NAMdPs structures were aligned (Figure 1). In order to better understand and visualize the structure–activity relationship (SAR), the amino acid residues (AA) of NAMdPs were denoted as AA-1 and AA-2, as shown in Figure 1. The alignment was done manually by using the Maestro program. In this way, we set the HSCH_2_–substituent and the amide groups as the backbone of the skeleton of peptides, and the substituents as AA-1 and AA-2, which were all different. When the AA-1 was proline, the HSCH_2_–groups were not typical because of the steric restrictions of the proline ring.

**Figure 2:**
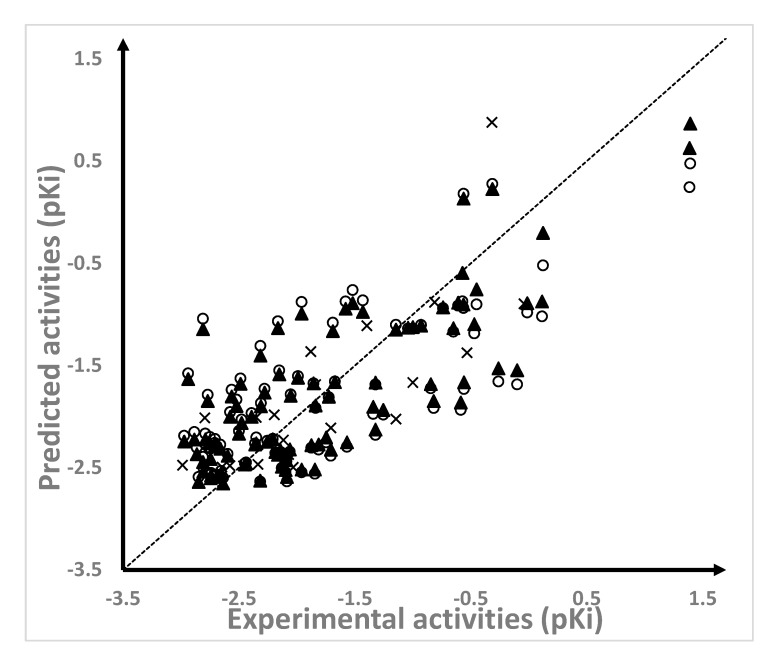
Scatter plots of the predicted versus experimental pKi values for the training set (▲), the cross-validated models over the training set (○), and the test set (×).

The field-based 3D-QSAR models were constructed by including the five available field descriptors in Phase (Phase, Schrodinger, LLC, New York, NY, USA, 2016) and by exploring the different number of principal components (PCs) through the partial least square (PLS) method. The best model, including two PCs, was statistically adequate: R^2^ = 0.617, standard deviation (SD) of 0.6, Q^2^ > 0.5 (Q^2^ = 0.529), and the stability was close to 1 (stability = 0.985). This model was also predictive, where R^2^_test_ > 0.6 (R^2^_test_ = 0.615) and standard deviation of the test set predictions (SD_test_) was 0.532. The predictions of the pKi values for the 95 compounds from the training set and the 23 compounds from the test set were reported in Table 1. The values of the predicted pKi values were plotted against the experimental values (Figure 2) for the training set, and the cross–validated model was plotted over the training set and the test set. As can be seen in Figure 2, the plotted predictions were well-distributed across the activity domain; although, the selected model seemed to have some problems in describing the SAR of the most active NAMdPs.

Although within the limit, our model complied with QSAR statistics defined by Golbraikh and Tropsha (Q^2^ > 0.5, R^2^_test_ > 0.6) [22]. We also performed further tests on the external validation, according to the Roy and Roy criteria [23]. This test was based on the following criteria for a QSAR model to have predictive power—(i) at least one of the correlation coefficients for regressions through the origin (predicted versus observed activities, or observed versus predicted activities), specifically: [(R^2^_test_ − R_0_^2^)/R^2^_test_] or [(R^2^_test_ − R′_0_^2^)/R^2^_test_] < 0.1; (ii) at least one slope (k or k′) of the regression lines through the origin should be close to 1, i.e., k or k′ should satisfy: 0.85 ≤ k ≤ 1.15, or 0.85 ≤ k′ ≤ 1.15; (iii) a high value of R^2^_m_ (R^2^_m_ > 0:5) was required, where R^2^_m_ = R^2^_test_ ×(1 − (R^2^_test_ − R_0_^2^)^1/2^. Our model complied with these criteria since [(R^2^_test_ − R_0_^2^)/R^2^_test_] = 0.03, k = 0.987, and R^2^_m_ = 0.530.

Despite the calculated statistic being adequate, the R^2^ and Q^2^ values did not represent the high-fitted rates, which was commonly associated to a lower QSAR predictive ability. However, this asseveration is a point of discussion in the literature [22,24,25]. For instance, in the paper ‘3D-QSAR illusions’ Doweyko [25] considered that a higher Q^2^ reflects that the model identified the redundancy in the training set and this has nothing to do with its predictability. Under this criterion, a low Q^2^ reflects that each member of the training set is important for the model. In any case, our purpose in this work was the interpretation of the model to describe the differential activity in the dataset, following the idea of Doweyko, which suggested the use of 3D-QSAR models as a retrospective analytical tool, instead a predictive tool [25].

**Figure 3:**
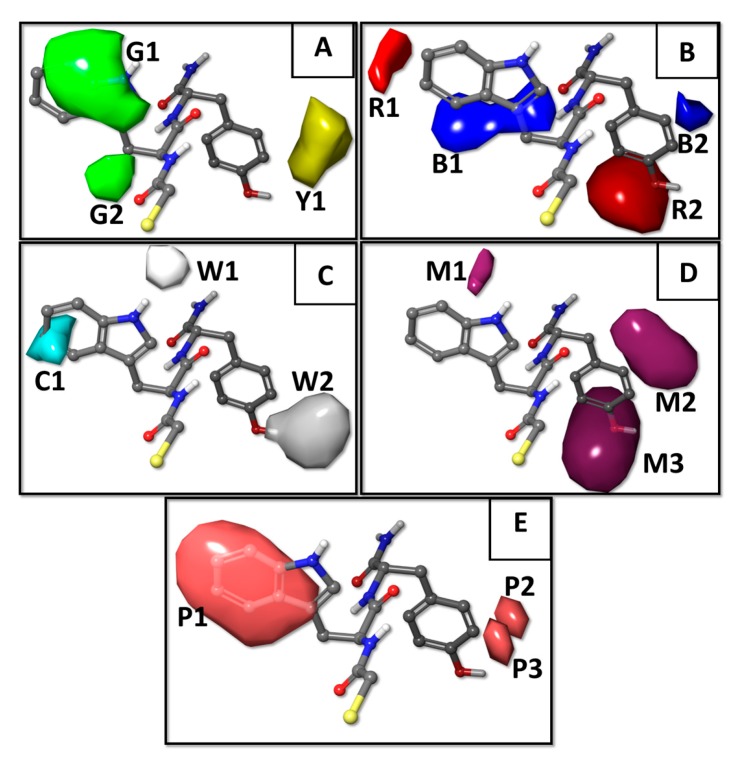
3D-QSAR model contour maps obtained with the five force fields and represented around the most active inhibitor in the dataset (compound **103**, HSCH_2_CO–Trp–Tyr–NH_2_). (**A**) Steric—the favorable and unfavorable components are in green (G1 and G2) and in yellow (Y1), respectively. (**B**) Electrostatic—the favorable and unfavorable components are in blue (B1 and B2) and in red (R1 and R2), respectively. (**C**) Hydrogen bond (HB)–donor—the favorable and unfavorable components are in white (W1 and W2) and in cyan (C1), respectively, and (**D**) HB–acceptor—the favorable components are in maroon (M1, M2, and M3). (**E**) Hydrophobic—the favorable components are in pink (P1, P2, and P3).

In the constructed 3D-QSAR model, the steric component had a 34.0% contribution, electrostatic had only 4.0%, hydrogen bond (HB)–donor had 13.4%, HB–acceptor had 21.9%, and hydrophobic had 26.7%. We utilized the contour isopleths projected in the most active NAMdP compound **103** (HSCH_2_CO–Trp–Tyr–NH_2_) to mechanistically interpret the best 3D-QSAR model and to predict the most favorable AA residues in each position AA-1 and AA-2. The five 3D-QSAR field contour plots are shown in Figure 3, labeled as (A) steric, (B) electrostatic, (C) hydrophobic, (D) hydrogen bond (HB)–acceptor, and (E) HB–donor.

The green (G1 and G2) and yellow (Y1) isopleths represent favorable and unfavorable components of the steric field (Figure 3A). Bulky groups were tolerated as AA-1 residue (green isopleths G1 and G2) to increase the inhibitory activity. Thus, NAMdPs with Trp, Phe, His, and Tyr in the side chain of AA-1 (isopleth G1) were among the most potent inhibitors in the dataset (considering pKi > −1.5 as a threshold, a deeper analysis revealed that 8 of 10 compounds with Trp as AA-1, 5 of 9 compounds with Phe as AA-1, 3 of 6 compounds with His as AA-1, and 8 of 9 compounds with Tyr as AA-1, were among the most potent LasB inhibitors). Additionally, the presence of alkyl substituents at the Cβ of AA-1 (isopleth G2), also increased the LasB inhibitory activity. In fact, the majority of NAMdPs increase their activities when Ala in AA-1 was replaced by Ile or Val residues. For instance, the pKi values increased when compounds **2**, **3**, and **6** (with AA-1 = Ala) were compared with **47**, **49**, and **54** (with AA-1 = Ile), respectively, and the same happened when compounds **1**, **4**, and **6** (with AA-1 = Ala) were compared with **114**, **116**, and **118** (with AA-1 = Val), respectively. Bulky substituents at the position AA-2 (yellow isopleth Y1) considerably decreased the biological activity. Thus, NAMdPs with Phe and Trp in the side chain of AA-2 (isopleth Y1) were among the less potent inhibitors in the dataset (considering pKi < −1.5 to be a threshold, a deeper analysis revealed that 11 of 15 compounds with Phe as AA-2 and 15 of 17 compounds with Trp as AA-2, were among the less potent LasB inhibitors).

Blue (B1 and B2) and red (R1 and R2) isopleths represent the favorable and unfavorable components of the electrostatic field. Blue isopleths were in the regions where the positive charges were favorable (or negative charges are unfavorable) for activity, and the red isopleths were in regions where more negative charges were favorable (or positive charges were unfavorable) for activity. Positively charged residues with a long side chain (e.g., Arg, Lys) as AA-1 and AA-2, had negative effects in the potency of the LasB inhibition (red isopleths R1 and R2 in Figure 3B). All compounds with Arg and Lys in AA-1 (isopleth R1) had pKi < −2.0 (Table 1). Meanwhile, NAMdPs with Arg and Lys in AA-2 (isopleth R2) were among the less potent inhibitors in the dataset (considering pKi < −1.5 as a threshold, a deeper analysis revealed that 11 of the 14 compounds with Arg as AA-2 and 13 of 18 compounds with Lys as AA-2, were among the less potent LasB inhibitors). On the other hand, negatively charged residues (e.g., Asp, Glu) as AA-1 and AA-2, had negative effects on the potency of LasB inhibition (blue isopleths B1 and B2 in Figure 3B). All compounds with Asp and Glu in AA-1 (isopleth B1) had pKi < −2.0 (Table 1). Meanwhile, NAMdPs with Asp in AA-2 (isopleth B2) were among the less potent inhibitors in the dataset (considering pKi < −1.5 as a threshold, a deeper analysis revealed that 6 of 8 compounds with Asp as AA-2 were among the less potent LasB inhibitors).

White (W1 and W2) and cyan (C1) isopleths represent the favorable and unfavorable components of the HB–donor field (Figure 3C). The white isopleth W1 indicates that Trp and His residues in AA-1 favor the inhibitory activity (considering pKi > −1.5 as the threshold, it is possible to see that 3 of 6 compounds with His as AA-1 and 7 of 10 compounds with Trp as AA-1, were among the more potent LasB inhibitors). Meanwhile, isopleth W2 indicated that Tyr and Gln residues in AA-2 were essential for increasing the inhibitory activity (all compounds with Tyr and Gln in AA-2 had pKi > −0.6). On the other hand, the cyan isopleth C1 indicated that the HB–donor groups in this region (provided by residues Arg, Lys, and Gln in AA-1) were unfavorable to perform a good inhibitory activity.

Maroon isopleths (M1, M2, and M3) represent the favorable components of the HB–acceptor field (Figure 3D). Isopleths M1 and M3 indicate that Tyr residue in AA-1 and AA-2, respectively, favor the LasB inhibitory activity (it is possible to see in Table 1 that the vast majority of compounds that contain tyrosine are potent inhibitors). On the other hand, isopleth M2 indicate that Gln residue in AA-2 favors the inhibitory activity (all compounds with Gln in AA-2 had pKi > −0.6).

Pink isopleths (P1, P2, and P3) represent the favorable components of the hydrophobic field (Figure 3E). These isopleths reflect that the presence of hydrophobic amino acids in AA-1 and AA-2 positions increases the LasB inhibition potency of NAMdPs. It is possible to see in Figure 3E that P1 encompasses the hydrophobic part of Trp in AA-1, and the side chain groups of Phe, Ile, Val, and Leu were also in this region. As mentioned above, there were instances of potent NAMdPs with these residues in AA-1. On the other hand, isopleths P2 and P3 indicate that the hydrophobic residues could increase the LasB inhibition potency. In fact, compounds with the residues Ile and Met had pKi > −0.7.

Thus, the 3D-QSAR let us conclude that the rational design of novel potential inhibitors should be directed to compounds that might have at least an aromatic and bulky group at the AA-1 position and a middle size with HB interactions at the AA-2 position. However, it is well-known that the 3D-QSAR models had limitations, since they did not consider protein–ligand interactions. To complement the 3D-QSAR results, in silico molecular docking experiments of all 118 NAMdPs were performed.

### 2.2. Molecular Docking Results

**Figure 4:**
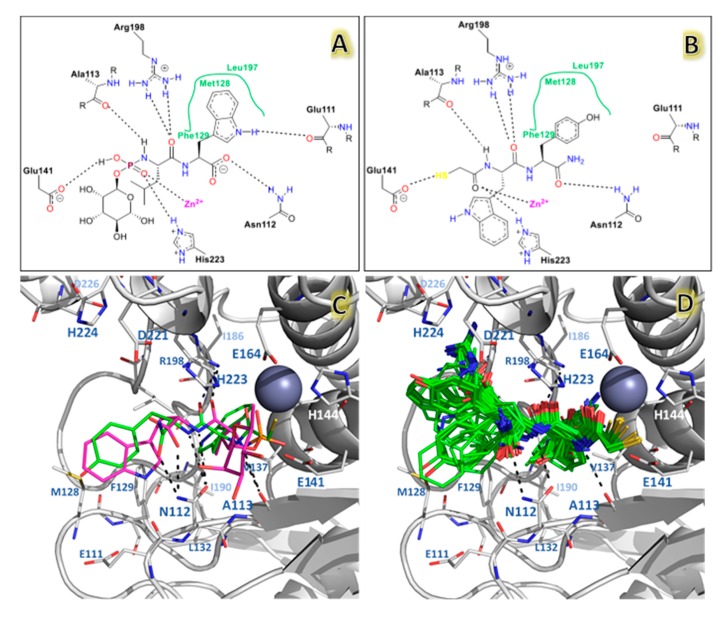
The docking results for NAMdPs and comparison with phosphoramidon in X-ray crystallographic structure 3DBK. Diagram for phosphoramidon (**A**), and the most active compound **103** (**B**) inside LasB binding site. (**C**) The docking pose obtained for compound **103** (green) and comparison with the crystallographic structures of phosphoramidon (purple). (**D**) The binding modes of the 118 compounds.

The docking method allows to create a protein–ligand interaction model for LasB inhibitors. The docking Glide scores of the selected poses per 118 ligands are reported in Table 1. These poses were first compared to the phosphoramidon inhibitor that was taken as a reference (Protein Data Bank (PDB) code 3DBK), since it was the only crystallized compound with a structure similar to that of NAMdPs. To get a better insight on the chemical environment surrounding the ligands, ligand interaction diagrams were sketched for phosphoramidon in the 3DBK crystal and the docking pose of the most active compound **103** (Figure 4).

The phosphate group of phosphoramidon in the crystallographic structure shows electrostatic interactions with the Zinc ion and the residue His223 of the LasB active site. The mercaptoacetyl group in all docked NAMdP poses was oriented to the same ion and histidine, in agreement with the previous report of Cathcart et al. [17]. These interactions keep the ligands fixed, allowing for better orientations of the whole structures to occupy the complete binding site. The backbone NH and CO groups of the AA-1 residue (Leu) in phosphoramidon and the AA-1 residues in 100% of the docked structures formed HB interactions with the residues A113 and R198. On the other hand, the backbone groups of the AA-2 residue (Trp) in phosphoramidon and the AA-2 residues of the docked structures formed HB interactions with the residues N112.

The docking poses of the entire set of 118 NAMdPs were compared with the conformation of phosphoramidon in the X-ray crystallographic structure (PDB: 3DBK). Figure 4c,d show that the docked structures fitted in an acceptable way with phosphoramidon. For a better insight, the above mentioned comparison was performed with an in-house script (named ligRMSD) [26], which identified the common graphs between molecules and calculated the root mean square deviation (RMSD) between the equivalent atoms of the common graphs. Since the NAMdPs were different from the reference compound (phosphoramidon), RMSD values were calculated by considering only the common graphs between molecules. In this context, %RefMatch and %MolMatch values were defined, where %RefMatch referred to the percent of common graphs between the docked compound and phosphoramidon, with respect to the total number of atoms of phosphoramidon; whereas, %MolMatch refers to the percent of common graphs between the docked compound, and phosphoramidon, with respect to the total number of atoms of the docked compound. These values allowed for identifying the maximal similitude between the docked compound and phosphoramidon; therefore, an RMSD value with high %RefMatch and %MolMatch values reflected that the compound under analysis bore a major resemblance with phosphoramidon.

The calculated RMSD values, reported in Table 1, were in the range of 0.4–3.1 Å. It is accepted in literature that RMSD = 2.0 Å could be considered to be the threshold value that discriminates between the right and wrong docking solutions for identical compounds [27,28] (this threshold could be higher for non-identical compounds).

Among the structures with lower RMSD values, a match that included just the peptide backbone was found, which is why the match percentage exhibited small values for that set. On the contrary, the NAMdPs with higher %MolMatch values (96%) included the entire set of HSCH2CO–(AA-1)–Trp–NH2 inhibitors, of which HSCH2CO–Leu–Trp–NH2, HSCH2CO–Asp–Trp–NH2, and HSCH2CO–Asn–Trp–NH2 showed better match values but were associated with high RMSD values (2.86–2.98 Å). These values reflected that the backbone atoms of the NAMdPs matched with the ones of phosphoramidon, but the side chain groups were differently oriented (Figure 4c,d). The NAMdP residues AA-2 contributed the most to increase the RMSD values. For instance, the side chain groups of the tryptophan residues for these inhibitors were rotated by some degrees with respect to this residue in phosphoramidon, due to the wide cavity of the binding pocket S2′. This higher flexibility led to higher RMSD values. The visual analysis of the structures (Figure 4d) indicated a similar orientation with respect to the reference phosphoramidon, which was reflected in the match between the backbone groups of both structures.

A systematic and detailed analysis of all possible interactions between LasB and the docked NAMdP could be performed by using Interaction Fingerprints (IFPs). Previous studies have demonstrated that IFP analysis is a valuable tool that allowed for a better schematization of protein–ligand interactions [26,29,30].

For a better comprehension of the interactions between the docked ligands and LasB, IFP analysis was performed. This analysis was a robust way of understanding the possible interactions between the receptor (enzyme) and the docked ligands. Then, the identification of those chemical interactions and the residues involved in them, could lead to a more detailed description of the LasB binding site available to the series of 118 NAMdP conformations, according to the docking results. This information could be considered for the design of novel potent inhibitors.

**Figure 5:**
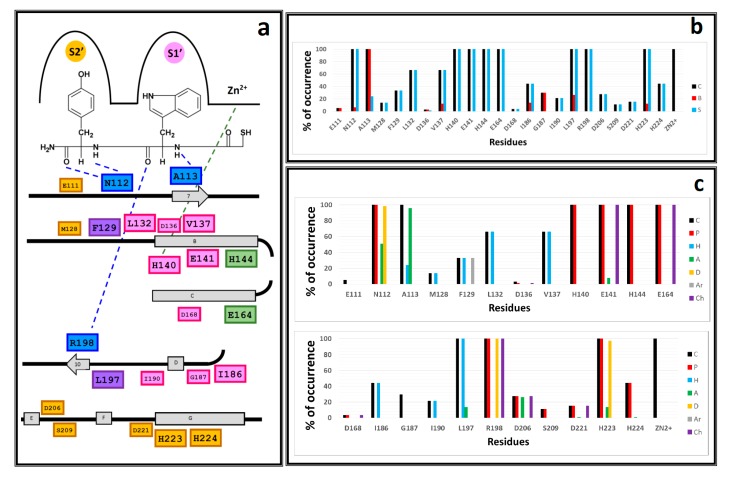
Occurrence of interaction types at the LasB–NAMdP binding interfaces. (**A**) Residues with observed interactions, their position in the LasB sequence, and their position in the binding site (with compound **103** as reference)—residues in pocket S1′ are in pink, residues in pocket S2′ are in orange, residues in the interface between the two pockets are in violet, residues coordinated to Zn^2+^ are in green, residues hydrogen-bonded to the backbone residues of the ligands are in blue. (**B**) Percentages of occurrence of contacts C, interactions with the backbone of the residue B, and interactions with the side chain of the residue S. (**C**) Percentages of occurrence of chemical interactions—contacts C, polar P, hydrophobic H, and HBs where the residue is acceptor A, HBs where the residue is donor D, aromatic Ar, and the electrostatic charged groups, Ch. The LasB–NAMdP structures obtained by docking were used for calculations of the percentages of occurrence represented here.

Plots of the chemical interactions types occurrence per residue are depicted in Figure 5. Residues with interactions and their position in the LasB sequence are depicted in Figure 5a. IFP analysis applied to the 118 LasB–NAMdP complexes is shown in Figure 5b,c. At first sight, there is a clear distinction between the residues belonging to both binding pockets previously described by Cathcart [17], called as S1′ (where AA-1 was placed) and S2′ (where AA-2 was placed). Topologically, the S1′ pocket was more internal and the S2′ pocket was formed by more superficial residues. Percent of occurrence plots showed that 23 residues of LasB were involved in the binding of NAMdPs.

IFPs reflected that 100% of the docked structures formed the above mentioned HB interactions with the residues A113 and R198 that anchored the backbone of the AA-1 ligand residues to the pocket S1′ and the HB interactions with the residue N112 that anchored the backbone of the AA-2 ligand residues to the pocket S2′. In this context, the plots in Figure 5c showed that A113 acted as the HB acceptor in more than 95% of the total structures, R198 acted as the HB donor and formed electrostatic interactions in 100% of the total structures, and N112 acted as the HB acceptor and HB donor in more than 50% and 98%, respectively, of the total structures. IFPs also showed that the residues H144 and E164, which were coordinated to Zn^2+^ and are close to the thiol groups of the docked NAMdPs, had polar contributions in 100% of the docked structures. E164 also had electrostatic contributions in 100% of them.

IFPs also reflected that the majority of the residues inside the pocket S1′ were mainly hydrophobic (Figure 5); in fact, the residues L132, V137, I186, I190, and L197 provide hydrophobic contributions to this pocket. L197 (also with some contacts in pocket S2′) showed these contributions in 100% of the docked poses. Additionally, L132 and V137 showed these contributions in more than 65% of the structures, and I186 and I190 showed them in more than 40% and 20% of the structures, respectively. It was noteworthy that I186 and I190 showed interactions with compounds that contained bulky AA-1 ligand residues such as Phe and Trp, and these residues were present in the AA-1 group of several of the most active NAMdPs. The residue E141, also in pocket S1′ and close to the mercaptoacetyl group, showed polar and electrostatic contributions in 100% of the docked structures, and the residue H140, which was also in pocket S1′ and was coordinated to Zn^2+^, showed polar contributions in 100% of the docked structures. Finally, the residue G187, also in pocket S1′, had contacts in around 30% of the docked structures.

Unlike pocket S1′, IFPs reflected that the pocket S2′ was more hydrophilic (Figure 5). The residues D206, S209, D221, H223, and H224 provided polar contributions to this pocket. The residue H223 had polar contributions in 100% of the docked poses. It also acted as the HB donor and the HB acceptor in more than 97% and 13% of them, respectively. The residue D206 had polar contributions and acted as the HB acceptor in more than 25% of the docked structures. The residues S209, D221, and H224 had polar contributions in 11.0%, 15.2%, and 44.0% of the docked structures. On the other hand, the residues M128 and F129, also in pocket S2′, provided hydrophobic contributions in 13.5% and 33.0% of the docked structures, respectively. F129, which also had some contacts in pocket S1′, showed aromatic contributions.

## 3. Materials and Methods

### 3.1. Dataset Collection and Pre-Processing

A dataset of 118 LasB inhibitors was collected from the two series reported by Cathcart et al. in publications [17,20]. Compounds with unknown activity values were excluded, and those with values reported in both papers kept the activity value with more precision (in all cases the pK_i_ values were virtually identical). K_i_ values were in the range of 4.05 × 10^−2^ *µ*M–971 *µ*M, and they were scaled using logarithmic scale as log (1/K_i_) (as pK_i_ units). The structures were pre-processed in Maestro using LigPrep (Maestro, Schrodinger LLC, New York, NY, USA, 2016), and the protonation states of titratable groups were predicted using Epik [31,32], at a pH value of 7.2. As the last preparation step, the molecular representations were visually inspected. The naming scheme of the ligands in terms of their two AA residues (i.e., AA-1 and AA-2 components) is represented in Table 1. The names are related to the position as well, such as a ligand with arginine as AA-1 and aspartic acid as AA-2 are represented as “HSCH_2_CO–Arg–Asp–NH_2_”.

### 3.2. QSAR Methodology

The 118 structures in the data set were aligned by hand in Maestro’s Molecular Editor (Maestro, Schrodinger LLC, New York, NY, USA). Moreover, the field-based 3D-QSAR models were trained in Phase (Phase, Schrodinger LLC, New York, NY, USA, 2016) by the random splitting implemented in this tool, getting a relation training/test sets of approximately 80/20 (95 and 23 compounds in training and test sets, respectively). The training process of 3D-QSAR models was carried out over the available descriptors, using the OPLS_2005 force field [33,34]. The fields were calculated on an orthohedral grid that enclosed the training set molecules, with a spacing of 1 Å and extended 3 Å beyond the limits of this set. The threshold for van der Waals and electrostatic interactions was set to 30 Kcal/mol, removing points closer than 2 Å to any of the training set atoms. During the PLS procedure, all variables (points in the grid) with a standard deviation of less than 0.01 were removed. Additionally, the variables whose regression coefficients were overly sensitive to small changes in the training set composition were removed using a |t–value| < 2.00 filter, as implemented in the Phase interface. Finally, the maximum number of PLS factors was set to 10.

### 3.3. Molecular Docking

Molecular docking calculations were performed using Glide [35] from the Schrodinger Suite. The coordinates of LasB were extracted from the PDB crystal with code 3DBK. This crystal is a complex between LasB and the ligand phosphoramidon. This ligand was similar in dimensions with the studied ligands; which is why it was used as a reference for the box generation. The downloaded crystal was pre-processed using the Protein Preparation Wizard (Protein Preparation Wizard, Schrodinger LLC, New York, NY, USA, 2016) protocol. The set of previously pre-processed ligands were docked in a 30 Å × 30 Å × 30 Å box, centered on the center of mass of phosphoramidon, covering the entire active site of the receptor. Standard (SP) and extra-precision (XP) modes were run in Glide [35], but only the XP mode was used to find adequate poses for all ligands [36]. The Glide protocol and parameters were the same as reported in previous reports [37,38,39]. The selection of poses was based on looking for the observed protein–ligand interactions patterns in the reported PDB crystals of LasB, and in the selection of the lowest scoring energy from among the adequate poses. Protein–ligand interaction patterns were identified and defined as essential chemical interactions described for analogue ligands (ECIDALs) [27,40] with LasB inhibitory activities. Finally, one pose per ligand was chosen.

### 3.4. IFP Calculations

IFPs defined in Singh reports [41,42] were calculated in Maestro (Maestro, Schrodinger LLC, New York, NY, USA, 2016) over the poses of ligands selected in docking experiments. The method identifies the presence of different chemotypes, such as polar (P), hydrophobic (H), HBs with an acceptor (A) as a residue group, HBs with a donor (D) as a residue group, aromatic (Ar), and electrostatic interactions with charged groups (Ch). These chemotypes were accounted as interactions between the ligands and the binding site residues of the target receptor. An interaction (under the chemotype definition) was accounted when a residue contained atoms within a specified cut-off distance from the ligand atoms.

## 4. Conclusions

Summing up, a set of 118 NAMdP inhibitors was studied by using 3D-QSAR modeling and molecular docking. QSAR analysis reflected that the side chain of the residue AA-1, at the NAMdP skeleton, should be mainly hydrophobic with bulky aromatic substituents (e.g., Phe, Trp, and Tyr). Furthermore, polar residues with large side chains (Lys, Arg, Glu, Gln, Asp) were not tolerated as AA-1. Meanwhile, non–bulky aromatic groups with functional groups were able to act, as HB donors are preferred as AA-2 (e.g., Gln and Tyr). The best compound in the studied set (compound **103**) had these preferred structural requirements to assure a good LasB inhibition—AA-1 was Trp, a bulky hydrophobic residue, and AA-2 was Tyr, which contained the hydroxyl group as an HB donor.

In addition, this work studied in detail the ligand–enzyme interactions of the whole set of compounds, and compared them with the ligand phosphoramidon (which is a crystal forming a complex with LasB). NAMdPs, according to the docking experiments, were oriented inside the LasB binding site by forming interactions with the Zn^2+^ ion, pocket S1′, and pocket S2′, as expected. The poses obtained by docking were used to carry out an IFP analysis, leading to a complete map of the LasB residues that interacted with NAMdPs.

The information provided here, through a combination of 3D-QSAR and docking experiments, might be taken into account by medicinal chemists interested in the synthesis and design of antimicrobials, specifically LasB inhibitors, with the goal of improving future rational drug design of specific potent therapeutics.

## Figures and Tables

**Table 1:** List of NAMdPs used in this study, their experimental and predicted pK_i_ values using the best QSAR model, their role in the QSAR construction (training or test set). Glide scores of their selected docking poses, and root mean square deviation (RMSD) values with respect to phosphoramidon in Protein Data Bank (PDB) structure 3DBK.

	NAMdP	Experimental pK_i_	Predicted pKi	QSAR Set	Glide Score (kcal/mol)	RMSD (Å)	%RefMatch	%MolMatch
1	HSCH_2_CO–Ala–Arg–NH_2_	−2.0607	−2.3215	Training	−7.652	0.95	38	67
2	HSCH_2_CO–Ala–Asp–NH_2_	−2.4997	−2.1689	Training	−5.239	0.95	38	78
3	HSCH_2_CO–Ala–Leu–NH_2_	−1.3222	−2.1287	Training	−5.067	0.96	38	78
4	HSCH_2_CO–Ala–Lys–NH_2_	−2.1847	−2.3482	Training	−5.975	0.94	38	74
5	HSCH_2_CO–Ala–Trp–NH_2_	−2.5966	−2.3854	Training	−5.161	3.09	62	96
6	HSCH_2_CO–Ala–Val–NH_2_	−1.7076	−2.1121	Test	−4.774	0.95	38	82
7	HSCH_2_CO–Arg–Asp–NH_2_	−2.8129	−2.3112	Test	−5.663	0.94	43	67
8	HSCH_2_CO–Arg–Lys–NH_2_	−2.1303	−2.4909	Training	−7.267	0.91	43	64
9	HSCH_2_CO–Arg–Phe–NH_2_	−2.3502	−2.2621	Training	−6.318	1.05	46	63
10	HSCH_2_CO–Arg–Trp–NH_2_	−2.0969	−2.5236	Training	−6.212	3.01	68	83
11	HSCH_2_CO–Asn–Arg–NH_2_	−2.4472	−2.4689	Training	−8.582	0.98	46	71
12	HSCH_2_CO–Asn–Leu–NH_2_	−2.7059	−2.2637	Training	−5.526	0.97	46	81
13	HSCH_2_CO–Asn–Lys–NH_2_	−2.4609	−2.4835	Test	−6.439	0.97	46	77
14	HSCH_2_CO–Asn–Phe–NH_2_	−1.5682	−2.2525	Training	−6.015	1.00	49	75
15	HSCH_2_CO–Asn–Trp–NH_2_	−1.8451	−2.5190	Training	−5.518	2.87	70	96
16	HSCH_2_CO–Asn–Val–NH_2_	−2.2553	−2.2475	Training	−5.682	0.99	46	85
17	HSCH_2_CO–Asp–Arg–NH_2_	−2.7412	−2.5774	Training	−8.351	0.98	46	71
18	HSCH_2_CO–Asp–Leu–NH_2_	−2.0719	−2.3721	Test	−5.413	0.95	46	81
19	HSCH_2_CO–Asp–Lys–NH_2_	−2.6911	−2.5914	Training	−5.397	0.96	46	77
20	HSCH_2_CO–Asp–Trp–NH_2_	−2.3160	−2.6275	Training	−4.564	2.86	70	96
21	HSCH_2_CO–Cys–Arg–NH_2_	−2.8102	−2.4454	Training	−5.857	1.18	41	68
22	HSCH_2_CO–Cys–Lys–NH_2_	−2.4378	−2.4601	Training	−3.691	1.18	41	75
23	HSCH_2_CO–Cys–Phe–NH_2_	−2.1173	−2.2302	Test	−3.155	0.97	43	73
24	HSCH_2_CO–Cys–Trp–NH_2_	−2.0334	−2.4966	Test	−3.091	3.05	65	96
25	HSCH_2_CO–Cys–Val–NH_2_	−2.2068	−2.2235	Training	−2.802	0.95	41	83
26	HSCH_2_CO–Gln–Arg–NH_2_	−2.3365	−2.4675	Test	−8.794	0.97	43	64
27	HSCH_2_CO–Gln–Leu–NH_2_	−2.7324	−2.2627	Test	−6.350	0.95	43	73
28	HSCH_2_CO–Gln–Lys–NH_2_	−2.5798	−2.4815	Test	−6.717	0.94	43	70
29	HSCH_2_CO–Gln–Trp–NH_2_	−1.9590	−2.5192	Training	−6.093	2.93	68	89
30	HSCH_2_CO–Gln–Val–NH_2_	−2.9717	−2.2451	Training	−6.254	0.93	43	76
31	HSCH_2_CO–Glu–Lys–NH_2_	−2.7427	−2.6041	Training	−5.895	0.93	43	70
32	HSCH_2_CO–Glu–Phe–NH_2_	−2.1644	−2.3753	Training	−4.813	1.15	46	68
33	HSCH_2_CO–Glu–Trp–NH_2_	−2.8476	−2.6418	Training	−5.828	2.97	68	89
34	HSCH_2_CO–Glu–Val–NH_2_	−2.8633	−2.3677	Training	−6.019	0.96	43	76
35	HSCH_2_CO–Gly–Arg–NH_2_	−2.8069	−2.5394	Training	−8.393	0.94	35	65
36	HSCH_2_CO–Gly–Leu–NH_2_	−2.1399	−2.3336	Training	−5.225	0.94	35	76
37	HSCH_2_CO–Gly–Lys–NH_2_	−2.6542	−2.5543	Training	−6.274	0.94	35	72
38	HSCH_2_CO–Gly–Phe–NH_2_	−1.7076	−2.3247	Training	−5.604	0.97	38	70
39	HSCH_2_CO–Gly–Trp–NH_2_	−2.0864	−2.5911	Training	−5.584	3.16	59	96
40	HSCH_2_CO–Gly–Val–NH_2_	−2.6599	−2.3163	Training	−5.022	0.94	35	81
41	HSCH_2_CO–His–Ala–NH_2_	−0.5587	−1.6658	Training	−3.155	0.93	38	70
42	HSCH_2_CO–His–Leu–NH_2_	−2.4857	−1.68	Training	−3.252	0.92	38	61
43	HSCH_2_CO–His–Lys–NH_2_	−2.5211	−1.8976	Training	−3.563	0.96	38	58
44	HSCH_2_CO–His–Phe–NH_2_	−1.3222	−1.6688	Training	−3.128	0.95	41	58
45	HSCH_2_CO–His–Trp–NH_2_	−1.2553	−1.9353	Training	−3.052	3.10	62	79
46	HSCH_2_CO–His–Val–NH_2_	−1.6721	−1.6632	Training	−2.932	0.94	38	64
47	HSCH_2_CO–Ile–Asp–NH_2_	−2.1523	−1.5871	Training	−4.167	1.00	43	76
48	HSCH_2_CO–Ile–Gln–NH_2_	−0.5717	−0.5951	Training	−6.036	0.92	43	73
49	HSCH_2_CO–Ile–Leu–NH_2_	−0.1004	−1.5472	Training	−4.684	0.94	43	76
50	HSCH_2_CO–Ile–Lys–NH_2_	−2.2788	−1.7662	Training	−5.633	0.95	43	73
51	HSCH_2_CO–Ile–Thr–NH_2_	−0.5328	−1.3756	Test	−6.040	0.93	43	80
52	HSCH_2_CO–Ile–Trp–NH_2_	−2.5635	−1.8041	Training	−5.822	2.92	68	93
53	HSCH_2_CO–Ile–Tyr–NH_2_	−0.3139	0.226	Training	−5.897	0.94	46	68
54	HSCH_2_CO–Ile–Val–NH_2_	−0.2625	−1.5298	Training	−4.851	0.93	43	80
55	HSCH_2_CO–Leu–Arg–NH_2_	−2.7945	−2.013	Test	−7.750	1.16	46	71
56	HSCH_2_CO–Leu–Asp–NH_2_	−2.7686	−1.8479	Training	−4.075	1.10	46	81
57	HSCH_2_CO–Leu–Leu–NH_2_	−1.7243	−1.8061	Training	−5.417	1.23	46	81
58	HSCH_2_CO–Leu–Lys–NH_2_	−1.1461	−2.0253	Test	−5.291	1.10	46	77
59	HSCH_2_CO–Leu–Phe–NH_2_	−2.0531	−1.7966	Training	−3.977	1.26	49	75
60	HSCH_2_CO–Leu–Trp–NH_2_	−2.4771	−2.0634	Training	−4.790	2.98	70	96
61	HSCH_2_CO–Lys–Asp–NH_2_	−2.9872	−2.4763	Test	−6.095	0.97	43	73
62	HSCH_2_CO–Lys–Leu–NH_2_	−2.0899	−2.4284	Training	−6.349	0.96	43	73
63	HSCH_2_CO–Lys–Lys–NH_2_	−2.6365	−2.6554	Training	−7.149	0.96	43	70
64	HSCH_2_CO–Lys–Phe–NH_2_	−2.1004	−2.4267	Training	−6.502	0.97	46	68
65	HSCH_2_CO–Lys–Val–NH_2_	−2.7443	−2.4166	Training	−6.072	0.96	43	76
66	HSCH_2_CO–Met–Arg–NH_2_	−0.8195	−1.8487	Training	−7.798	1.00	43	67
67	HSCH_2_CO–Met–Asp–NH_2_	−0.8451	−1.6836	Training	−4.891	0.99	43	76
68	HSCH_2_CO–Met–Lys–NH_2_	−0.5866	−1.8627	Training	−6.684	0.97	43	73
69	HSCH_2_CO–Met–Phe–NH_2_	−2.9380	−1.6339	Training	−5.564	1.19	46	71
70	HSCH_2_CO–Met–Trp–NH_2_	−2.3096	−1.9004	Training	−4.916	2.89	68	93
71	HSCH_2_CO–Met–Tyr–NH_2_	−0.5623	0.1316	Training	−5.955	1.26	46	68
72	HSCH_2_CO–Met–Val–NH_2_	−1.9912	−1.6213	Training	−5.815	0.97	43	80
73	HSCH_2_CO–Phe–Gln–NH_2_	0.1226	−0.2028	Training	−6.245	0.96	38	56
74	HSCH_2_CO–Phe–Ile–NH_2_	−0.6503	−1.1362	Training	−5.120	0.93	38	58
75	HSCH_2_CO–Phe–Leu–NH_2_	−2.8096	−1.146	Training	−4.450	0.95	38	58
76	HSCH_2_CO–Phe–Lys–NH_2_	−1.8808	−1.3642	Test	−5.413	0.95	38	56
77	HSCH_2_CO–Phe–Met–NH_2_	−0.4502	−0.7559	Training	−6.914	0.91	38	58
78	HSCH_2_CO–Phe–Phe–NH_2_	−2.1644	−1.1354	Training	−5.606	1.23	41	56
79	HSCH_2_CO–Phe–Trp–NH_2_	−2.3139	−1.4029	Training	−7.568	1.76	62	77
80	HSCH_2_CO–Phe–Tyr–NH_2_	1.3872	0.6276	Training	−6.918	0.95	41	54
81	HSCH_2_CO–Phe–Val–NH_2_	−1.0414	−1.1315	Training	−4.537	0.96	38	61
82	HSCH_2_CO–Pro–Arg–NH_2_	−1.7482	−2.2055	Training	−7.338	1.53	38	73
83	HSCH_2_CO–Pro–Leu–NH_2_	−2.3909	−2.0021	Training	−7.612	1.40	38	85
84	HSCH_2_CO–Pro–Lys–NH_2_	−2.8842	−2.2260	Training	−8.277	1.75	38	81
85	HSCH_2_CO–Pro–Trp–NH_2_	−2.7497	−2.2623	Training	−5.630	2.96	62	96
86	HSCH_2_CO–Pro–Val–NH_2_	−2.1959	−1.9837	Test	−7.628	1.48	38	92
87	HSCH_2_CO–Ser–Arg–NH_2_	−2.6474	−2.4949	Test	−8.952	0.94	41	68
88	HSCH_2_CO–Ser–Leu–NH_2_	−2.7076	−2.2896	Training	−6.262	1.14	41	79
89	HSCH_2_CO–Ser–Phe–NH_2_	−1.8751	−2.2801	Training	−6.316	1.15	43	73
90	HSCH_2_CO–Ser–Val–NH_2_	−2.3598	−2.2737	Training	−5.362	1.12	41	83
91	HSCH_2_CO–Thr–Arg–NH_2_	−2.7896	−2.2227	Training	−9.096	1.07	41	65
92	HSCH_2_CO–Thr–Phe–NH_2_	−2.3522	−2.0078	Test	−5.473	1.13	43	70
93	HSCH_2_CO–Thr–Trp–NH_2_	−1.8129	−2.2743	Training	−6.166	2.99	65	92
94	HSCH_2_CO–Thr–Val–NH_2_	−2.5763	−2.0035	Training	−5.808	1.06	41	79
95	HSCH_2_CO–Trp–Arg–NH_2_	−1.3979	−1.1114	Test	−8.417	0.94	38	47
96	HSCH_2_CO–Trp–Asp–NH_2_	−1.5798	−0.9463	Training	−6.044	0.96	38	52
97	HSCH_2_CO–Trp–Glu–NH_2_	−1.9590	−0.9930	Training	−6.483	0.99	38	50
98	HSCH_2_CO–Trp–Ile–NH_2_	−0.0128	−0.8916	Training	−7.636	0.51	38	52
99	HSCH_2_CO–Trp–Leu–NH_2_	−0.5635	−0.9039	Training	−5.190	0.95	38	52
100	HSCH_2_CO–Trp–Lys–NH_2_	−1.0000	−1.1254	Training	−8.613	0.66	38	50
101	HSCH_2_CO–Trp–Phe–NH_2_	−0.0414	−0.8966	Test	−6.127	1.04	41	50
102	HSCH_2_CO–Trp–Trp–NH_2_	−1.6902	−1.1641	Training	−5.961	3.04	62	70
103	HSCH_2_CO–Trp–Tyr–NH_2_	1.3925	0.8661	Training	−5.518	0.98	41	48
104	HSCH_2_CO–Trp–Val–NH_2_	−0.6096	−0.8870	Training	−5.893	0.98	38	54
105	HSCH_2_CO–Tyr–Arg–NH_2_	−0.4698	−1.0968	Training	−10.083	0.59	38	50
106	HSCH_2_CO–Tyr–Asp–NH_2_	−0.7404	−0.9317	Training	−6.700	0.43	38	56
107	HSCH_2_CO–Tyr–Glu–NH_2_	−1.4314	−0.9771	Training	−7.712	0.47	38	54
108	HSCH_2_CO–Tyr–Leu–NH_2_	−1.5185	−0.8919	Training	−7.353	0.43	38	56
109	HSCH_2_CO–Tyr–Lys–NH_2_	−0.9294	−1.1111	Training	−8.097	0.52	38	54
110	HSCH_2_CO–Tyr–Phe–NH_2_	−0.8129	−0.8823	Test	−7.380	0.50	41	54
111	HSCH_2_CO–Tyr–Trp–NH_2_	−1.1461	−1.1498	Training	−7.392	2.81	62	74
112	HSCH_2_CO–Tyr–Tyr–NH_2_	−0.3181	0.8790	Test	−7.686	0.87	41	52
113	HSCH_2_CO–Tyr–Val–NH_2_	0.1146	−0.8746	Training	−7.274	0.46	38	58
114	HSCH_2_CO–Val–Arg–NH_2_	−1.8388	−1.8918	Training	−8.183	1.00	41	65
115	HSCH_2_CO–Val–Leu–NH_2_	−1.8388	−1.6874	Test	−5.767	0.94	41	75
116	HSCH_2_CO–Val–Lys–NH_2_	−1.3424	−1.9057	Training	−5.501	0.95	41	71
117	HSCH_2_CO–Val–Phe–NH_2_	−1.8573	−1.6755	Training	−5.001	0.95	43	70
118	HSCH_2_CO–Val–Val–NH_2_	−1.0000	−1.6676	Test	−5.225	0.95	41	79

## Abbreviations

3D	three-dimensional
AA	amino acid
HB	Hydrogen bonding
IFPs	interaction fingerprints analysis
LasB	*Pseudomona* elastase
NAMdP	*N*-alpha-mercaptoacetyl dipeptide
PDB	Protein Data Bank
RMSD	root mean square deviation
SAR	structure–activity relationship
